# Interaction between Melatonin and NO: Action Mechanisms, Main Targets, and Putative Roles of the Emerging Molecule NOmela

**DOI:** 10.3390/ijms23126646

**Published:** 2022-06-14

**Authors:** Sara E. Martínez-Lorente, Miriam Pardo-Hernández, José M. Martí-Guillén, María López-Delacalle, Rosa M. Rivero

**Affiliations:** 1 Center of Edaphology and Applied Biology of Segura CEBAS-CSIC, Campus Universitario Espinardo, 30100 Murcia, Spain; martsara999@gmail.com (S.E.M.-L.); mpardo@cebas.csic.es (M.P.-H.); jmarti@cebas.csic.es (J.M.M.-G.); mlopez@cebas.csic.es (M.L.-D.); 2 Faculty of Biology, Department of Plant Physiology, University of Murcia, Campus Universitario Espinardo, 30100 Murcia, Spain

**Keywords:** melatonin, NO, ROS, abiotic stress, PTMs, H_2_S, RNS, NOmela

## Abstract

Melatonin (MEL), a ubiquitous indolamine molecule, has gained interest in the last few decades due to its regulatory role in plant metabolism. Likewise, nitric oxide (NO), a gasotransmitter, can also affect plant molecular pathways due to its function as a signaling molecule. Both MEL and NO can interact at multiple levels under abiotic stress, starting with their own biosynthetic pathways and inducing a particular signaling response in plants. Moreover, their interaction can result in the formation of NOmela, a very recently discovered nitrosated form of MEL with promising roles in plant physiology. This review summarizes the role of NO and MEL molecules during plant development and fruit ripening, as well as their interactions. Due to the impact of climate-change-related abiotic stresses on agriculture, this review also focuses on the role of these molecules in mediating abiotic stress tolerance and the main mechanisms by which they operate, from the upregulation of the entire antioxidant defense system to the post-translational modifications (PTMs) of important molecules. Their individual interaction and crosstalk with phytohormones and H_2_S are also discussed. Finally, we introduce and summarize the little information available about NOmela, an emerging and still very unknown molecule, but that seems to have a stronger potential than MEL and NO separately in mediating plant stress response.

## 1. Introduction

N-acetyl-5-methoxytryptamine, better known as melatonin (MEL), is an indole-derived compound similar to indole-3-acetic acid (IAA). MEL is an indolamine, and although it was first discovered in bovine extracts from the pineal gland in 1958, it was only isolated and identified in 1960 by Lerner et al. [1]. It was named after its ability to aggregate melanin granules in skin chromatophores. Although it was believed that this compound was only present in animals, in 1995, two independent groups identified the presence of MEL in higher plants [2,3]. In the following years, it was proven that MEL was also found in several Eukarya and Bacteria groups, although there is still no evidence for its presence in the Archaea domain. Due to its ubiquitous distribution, it has been suggested that the structure of this molecule has barely changed through evolution [4,5]. Initially, this molecule acted as an antioxidant in unicellular organisms, but through evolution, this role changed, and it started to act as a hormone in superior eukaryotes, being involved in diverse processes such as immunomodulation, circadian rhythms, or seasonal reproductive regulation [6].

In 2004, the term phytomelatonin was proposed to discriminate between animal MEL and plant MEL [7]. Apart from regulating plant growth, in the last few years, it has been described as a “master regulator” involved in plant cell metabolism, regulating and increasing plant tolerance to biotic and abiotic stress. This is possible due to its ability to act as a hormone or as an antioxidant molecule, by scavenging diverse reactive oxygen species (ROS) and reactive nitrogen species (RNS) [8,9,10]. In addition, MEL can easily pass through cell membranes and move in the cytosol and organelles due to its amphiphilic and amphipathic character [11], thus facilitating its regulatory role in plant metabolism.

Gasotransmitters are molecules involved in the regulation of plant development and stress responses. The principal gasotransmitters are nitric oxide (NO), carbon monoxide (CO), and hydrogen sulfide (H_2_S). NO is a potent signaling molecule due to its short half-life and high diffusibility across the plasma membrane. It is a molecule with dual functions, as it can act both as an antioxidant or pro-oxidant, depending on its concentration, with important roles in flowering, plant growth, morphogenesis regulation, and oxidative stress response [12]. The interaction of NO with target molecules results in the production of RNS, and although RNS implication in cell metabolism still needs further research [13], some known RNS aspects are described in the present review. Both NO and MEL can interact at multiple levels, modulating cell metabolism during plant development, although some aspects of the routes through which this interaction occurs have not been described yet. Given the importance of these two molecules (i.e., NO and MEL), and their interaction in plant physiology and plant stress response, this review focuses on the role and the putative and known interactions between NO and MEL in different contexts.

## 2. Interaction between MEL and NO Biosynthetic Pathways: Synergistic and Antagonistic Interactions

**Figure 1:**
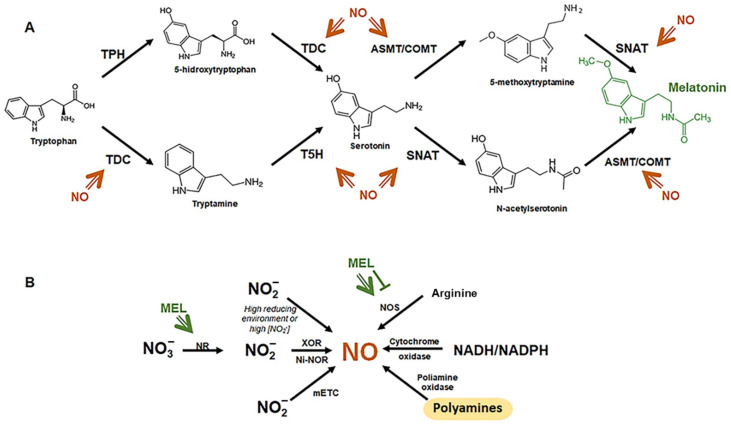
Melatonin (**A**) and NO (**B**) biosynthetic pathways. The figure shows the specific points in the melatonin biosynthetic pathway that NO is able to regulate, and vice versa, through the modification of key enzymes within each pathway.

MEL’s precursor is tryptophan, an amino acid that plants can synthetize through the shikimate pathway (which is also the synthesis route for all aromatic amino acids in plants). First, tryptophan is converted to serotonin through two different pathways: tryptophan’s decarboxylation into tryptamine via tryptophan decarboxylase (TDC), followed by its hydroxylation into serotonin by tryptamine 5-hydroxylase (T5H); or tryptophan hydroxylation into 5-hydroxytryptophan by tryptophan hydroxylase (TPH), followed by its decarboxylation into serotonin by TDC, with the first route being most frequent in plants. Once serotonin is produced, it is acetylated to N-acetylserotonin by serotonin N-acetyltransferase (SNAT), which is finally methylated by O-methyltransferase (ASMT), although this last step can also be performed by caffeic acid 3-O-methyltransferase (COMT). There is also an alternative pathway that leads to MEL formation, the first step of which is serotonin catalysis to 5-methoxytryptamine by ASMT or COMT, which is converted to MEL by SNAT (Figure 1A). Depending on the route followed, MEL can be synthesized in the cytoplasm or the chloroplast, respectively [14,15]. MEL can also generate active derivatives under physiological conditions. Specifically, 2-hydroxymelatonin (2OHM) is the most abundant in plants, and some authors affirm that it induces stress tolerance in plants more effectively than MEL [16,17]. Plants vary in their MEL levels, from high to undetectable concentrations [11], underlining that stress conditions enhance MEL production [4,18]. Within plants, it has been reported that flowers have the highest MEL levels, followed by leaves and seeds, which can be explained by the need to maintain a high antioxidant environment in flowers and leaves, as these organs are more exposed to stress conditions [4]. MEL is mainly synthetized in chloroplasts and mitochondria, which correlates with its protective role against free radicals produced in these organelles [14,19]. At basal conditions, both ROS and MEL are kept at relatively low and constant levels, with their synthesis being upregulated under stress conditions [20].

NO production can occur via oxidative and reductive pathways. NO is synthetized through the oxidative pathway via the oxidation of L-arginine by NO synthase (NOS), polyamine by polyamine oxidase, or NADH/NADPH via cytochrome oxidase. Meanwhile, the reductive pathway is characterized by the reduction of NO_3_^−^ to NO_2_^−^ by nitrate reductase (NR), using NADH as the electron donor, under anoxic conditions. This is followed by NO_2_^−^ reduction to NO due to the action of xanthine oxidoreductase (XOR), and plasma-membrane-bound nitrite–NO reductase (Ni-NOR). Moreover, NO can be generated within polyamine metabolism. High nitrate concentrations or highly reducing environments trigger the non-enzymatic reduction of nitrite to NO. The mitochondrial electron transport chain, under acidic and anoxic conditions, can also produce NO from nitrite (Figure 1B) [21,22,23,24,25,26]. Due to the short half-life of NO (~30 s), NO transport and accumulation in plants are mediated by NO carriers (which are more stable in solution), mainly S-nitrosothiols [27]. S-nitrosothiols (SNOs) are non-protein sulfhydryl-containing compounds that are formed by their reaction with NO. They are more stable than NO and can be transported and act as NO storage. In addition, SNOs can carry out post-translational modifications (PTMs) mediated by cell signaling, especially during the stress response, as they can act as S-nitrosylating agents, which can react with thiol groups [28]. SNOs can be classified by their molecular mass into high-molecular-mass S-nitrosothiols (HMM-SNOs) or low-molecular-mass S-nitrosothiols (LMM-SNOs), with GSNO (S-nitrosoglutathione) being the most abundant LMM-SNO in biological systems, being generated via NO interaction with reduced glutathione [29]. GSNO constitutes a NO reservoir that can be degraded by S-nitrosoglutathione reductase (GSNOR). Lee et al. [30] showed that plant development and stress response were defective after mutations in the *GSNOR* gene, confirming NO role in plant metabolism.

In recent years, the crosstalk between NO and MEL in plant physiology has been described, as MEL can affect endogenous NO levels and NO can alter endogenous MEL content (Figure 1). MEL can either induce NO production or scavenge NO and is also capable of increasing NO levels by upregulating *NOS* gene expression [31,32]. Experiments performed in tomato seedlings showed that exogenous MEL inhibits S-nitrosoglutathione reductase (GSNOR) activity and upregulates NR activity, which elevates endogenous NO levels [33]. On the other hand, NO can also promote MEL biosynthesis by inducing the expression of *TDC*, *T5H*, *SNAT*, and *COMT* genes, which code for the main enzymes found in the MEL biosynthesis pathway. In addition, NO also increases MEL levels by modulating the activity of MEL synthesis enzymes [8,34].

The crosstalk between NO and MEL is especially relevant during the plant’s response to unfavorable situations. Under stress conditions, MEL triggers NO accumulation by modulating the activity of NR and NOS via the arginine pathway, as well as the expression of related genes [31,35]. However, NO and MEL also have antagonistic actions. MEL can inhibit NOS, decreasing NO levels, through various mechanisms [36], but it can also promote NO accumulation via the arginine pathway [31] or upregulate NOS-related genes by increasing NOS activity and, thereby, NO levels [32].

These kinds of interactions between these two molecules, and the described fine-tuned regulation exerted by both molecules on each other, deserve further investigation to discover the specific cell signaling processes that govern the plant’s adaptation to climate change.

## 3. MEL and NO Action on Plant Growth and Development: Physiological Responses and Effect on Fruit Ripening

Both MEL and NO play fundamental roles during plant development, as they modulate several essential processes such as plant growth, senescence, flowering, and fruit ripening [37,38,39]. As a consequence, it is of vital importance to understand how these molecules help the regulation of plant and fruit developmental processes.

### 3.1. Plant Development

The first phytomelatonin receptor, CAND2/PMRT1, was recently discovered and identified in *Arabidopsis* by Wei et al. [40]. Among its functions, it was shown that the union between CAND2/PMRT1 and MEL can control stomatal closure via the Ca^2+^ and H_2_O_2_ signaling cascade. MEL binding to this receptor activates G_γb_ dissociation from G_α_, triggering H_2_O_2_ production by NADPH-oxidase, which enhances Ca^2+^ influx and K^+^ efflux, causing stomatal closure [40].

In plants, MEL is involved in plant development and growth, due to its action as an auxin-like molecule [41]. MEL is not capable of stimulating IAA synthesis, but it can affect plant growth in an auxin-independent manner (as it does not activate the expression of *DR5:GUS*, an auxin-inducible gene marker, and there is no evidence that MEL can be perceived by auxin receptors), although initially MEL was described as an auxin-mimetic molecule [42,43]. It is involved in processes such as flowering, leaf senescence, root morphogenesis, and fruit ripening, as well as the modulation of chlorophyll and proline levels in leaves and fruits [4,42,44,45,46,47]. In recent studies by Lee and Back (2019), mutant plants, in which *SNAT* was downregulated, showed a semi-dwarf stature, which confirmed the action of MEL as a growth promoter [48].

Arnao et al. [42] demonstrated the role of MEL in improving rhizogenesis. Lately, it was shown that exogenous MEL promoted lateral and adventitious root formation in *Lupinus albus* rice, cucumber, and Arabidopsis [42,43,49,50]. Moreover, MEL was proven to modulate root gravitropic response [51]. Exogenous MEL may either promote or inhibit plant growth depending on its concentration, with a higher inhibitory effect at higher concentrations, due to its auxin-like effects. It has been demonstrated that high MEL concentrations (100 µM) inhibit root growth, while low MEL concentrations (0.1 µM) promote it, at the same time inducing an increase in endogenous IAA levels (it is thought that this IAA increase triggers root growth) [52]. Thus, high MEL concentrations are associated with a decrease in IAA biosynthesis, reducing root meristem size [53]. In addition, it has been proven that the genes regulated by low MEL concentrations are different from the ones regulated at high MEL concentrations [54]. In some plant species such as cucumber, exogenous MEL did not affect the expression of auxin-related genes [50], although experiments performed in Arabidopsis showed that high MEL concentrations downregulated the expression of genes encoding auxin-influx carrier proteins (AUX1/LAX) [54] and auxin biosynthesis [53].

Similarly, NO is capable of regulating auxin responses, promoting adventitious root formation, as well as lateral roots growth, root hair development, and root gravitropism. [55,56]. Specifically, it has been suggested that NO can also regulate root growth via the cGMP (cyclic guanosine monophosphate) pathway, acting as a second messenger in this route [57]. An increase in NO levels inhibited root meristem activity and reduced the number of dividing cells in primary roots by inhibiting auxin transport and response owing to the downregulation of auxin efflux protein PIN-FORMED 1 (PIN1), as shown by Fernández-Marcos et al. [58]. Thus, the effects of high NO levels are similar to the ones observed by high MEL levels. Another mechanism of NO action is characterized by the capacity of NO to induce protein PTMs, mainly S-nitrosylation, which can modulate enzyme activity and protein function, although its relationship with plant development requires further research [59]. In the last few years, some researchers have shown an interesting interaction between MEL and NO. In this regard, Wen et al. [33] showed that in tomato plants, exogenous MEL triggered NO production by promoting NR activity, which induced the formation of adventitious roots through the modulation of the expression of auxin-related genes, such as the genes involved in auxin accumulation, transport, and signal transduction. Moreover, MEL is capable of regulating the NO/NOS system in order to perform physiological functions [60]. Likewise, MEL synthesis was shown to be induced by NO exogenous treatment in tomato seedlings [41], which also induced root development, indicating that there may be a feedback loop between NO and MEL that influences root development via auxin signaling pathways.

MEL also plays a role in regulating floral transition. Shi et al. [61] showed that MEL mediated the stabilization of DELLA proteins, which disturbed flowering-related transcription factors, thus repressing the floral transition. On the other hand, an excess of exogenous MEL triggered the activation of Flowering Locus C (*FLC*), thus delaying flowering. However, strigolactone (a carotenoid-derived phytohormone) can act upstream of MEL, inducing floral transition by inhibiting MEL synthesis [62]. Lozano-Juste and León [63] showed that NO can also disturb flowering by increasing DELLA protein levels. Due to this, it has been hypothesized that flowering is regulated by a NO/MEL crosstalk, although the mechanisms involved in this process are still unknown, and more research is needed on this putative crosstalk hypothesis.

MEL has been reported to have antisenescence proprieties, which have been attributed to its role as an antioxidant. Moreover, MEL can impede the upregulation of *Hexokinase-1*, a senescence-associated gene, and autophagy-related genes (*ATGs*), thereby preventing leaf senescence [64,65,66]. In apples, during leaf senescence, MEL is capable of preventing carotenoid and chlorophyll degradation. Moreover, exogenous MEL increased fructose, sorbitol, sucrose, glucose, and starch levels, and prevented the decline of Rubisco and soluble protein content. [66,67]. However, NO regulation during these processes is still unknown.

### 3.2. Fruit Ripening

Fruit ripening is defined as a complex process, orientated toward promoting animal-mediated seed dispersion, which involves changes in fruit organoleptic proprieties. During the ripening of climacteric fruits, both MEL and NO have been shown to inhibit ethylene biosynthesis, preventing postharvest senescence. During fruit ripening, NO can interact with ACC oxidase, a key enzyme in ethylene biosynthesis, generating an ACC oxidase–NO complex, which can also form a stable ternary ACC–ACC oxidase–NO complex via chelation by ACC. This metabolic step decreases ethylene production by inactivating ACC oxidase [68]. Moreover, NO can nitrosate ACO (also involved in ethylene production), decreasing its activity and downregulating ACO gene expression (*LeACO1*, *LeACOH2*, and *LeACO4*), thus reducing ethylene levels [69].

Soluble sugars also play important roles in fruit ripening, as they can act as signaling molecules and participate in mediating fruit ripening and senescence. Shi et al. [70] showed that exogenous NO-treatment-modulated sugar metabolism by enhancing sucrose phosphate synthase (SPS), sucrose synthase (SS), and neutral invertase (NI) activity, thereby maintaining higher sucrose, fructose, and glucose levels. Thus, NO treatment acts by inhibiting ethylene biosynthesis, as well as by regulating sugar metabolism in postharvest fruit programs [71].

Sun et al. [72] showed that, in tomatoes, MEL stimulated fruit ripening by upregulating the expression of ethylene-signal-transduction-related genes, thus inducing ethylene production. Moreover, it has been shown that MEL increases ripening- and anthocyanin-increase-related protein levels [73]. This results in amplification in ethylene signal transduction, which triggers cell wall degradation, lycopene accumulation, and the synthesis of volatile organic compounds (VOCs) via gene regulation [74]. However, in some fruits, such as bananas, exogenous MEL repressed ethylene synthesis, thus delaying the ripening process [75]. In tomatoes, MEL acts as an antioxidant molecule that scavenges RNS during fruit ripening and enhances arginine-pathway-mediated NO accumulation, as well as polyamines and proline production. On the other hand, in pears, in order to delay postharvest senescence, MEL was capable of reducing ethylene production by regulating the synthesis of NO. The inhibition of NO synthesis eliminated the effect of MEL delaying fruit ripening, which may indicate that MEL acts upstream of NO in this pathway [13,31,32,68,76,77]. Thereby, MEL does not directly repress ethylene biosynthesis; it inhibits ethylene production via NO-mediated mechanisms.

## 4. MEL and NO Interaction during Molecular Metabolic Regulation under Abiotic Stress

In plant physiology, stress is defined as a condition that prevents normal development, growth, and metabolism [78]. Climate change is likely to increase the impact of stress factors on plants, which can limit plant production in the following years [79], thus representing a major challenge for agricultural lands.

**Figure 2:**
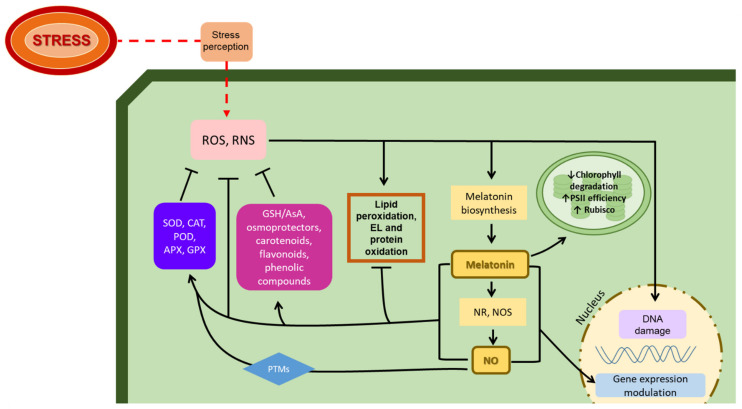
General mechanisms for MEL- and NO-mediated stress response. Stress causes an increase in ROS and RNS levels, which damage plant cells. Increased ROS levels trigger MEL production, which can also promote NO synthesis. Both MEL and NO directly scavenge ROS and RNS and promote the activity of antioxidant enzymes, the accumulation of antioxidant molecules, and osmoprotectants, and influence gene expression, thus alleviating the effects of stress on cells.

Abiotic stresses can affect plant growth and development, as well as reproductive programs. However, plants can adapt to environmental stresses through complex mechanisms involving changes in enzyme activity, gene expression, and the accumulation of key molecules. Under stress conditions, there is an immediate response characterized by an increase in ROS, RNS, and malondialdehyde (MDA) levels. High concentrations of ROS and RNS may lead to membrane damage due to lipid peroxidation and electron leakage (EL), but they can also cause DNA damage, impaired enzyme activity, and carbohydrate oxidation [80] (Figure 2). ROS, such as superoxide anion, hydrogen peroxide, or hydroxyl radical, are continuously produced in plants and can act as signaling molecules, although high ROS levels can lead to a situation of oxidative stress. Due to this, plants have developed both enzymatic and non-enzymatic antioxidant systems in order to protect themselves against oxidative stress. Enzymatic systems include enzymes such as superoxide dismutase (SOD), catalase (CAT), peroxidase (POD), glutathione reductase (GR), glutathione peroxidase (GPX), etc. Likewise, non-enzymatic systems are constituted by antioxidant molecules such as glutathione (GSH), ascorbic acid (AsA), flavonoids, or carotenoids. As is well-known, ROS can activate numerous stress response pathways, and among them, ROS upregulate MEL biosynthesis, which acts by balancing redox homeostasis either directly by scavenging free radicals, or indirectly by increasing the activity of key antioxidant enzymes (SOD, CAT, POD, APX, and/or GPX). Moreover, MEL can also increase the concentration of GSH and AsA [34,37,81,82,83,84] (Figure 2). On the other hand, MEL acts by decreasing EL and MDA levels, thus alleviating abiotic-stress-related membrane damage [85,86]. MEL also increases the transcription of many stress-tolerance-related genes and activates several downstream signaling transduction pathways [87] (Figure 2).

In terms of photosynthetic efficiency, MEL reduces chlorophyll degradation, thus improving photosynthetic efficiency during stress conditions [88]. It also regulates the accumulation of key proteins, such as 1,5 bisphosphate carboxylase/oxygenase (Rubisco), and improves the efficiency of photosystem II reaction centers [64,88,89]. Another significant aspect is that MEL increases leaf area, which also helps to improve the photosynthesis rate [83] (Figure 2). Wei et al. [90] showed that MEL upregulated the expression of ferredoxin, photosystem I subunits (PsaK and PsaG), and photosystem-II-related elements (PsbO and PsbP), and is described by these authors as a key molecular regulator of the photosynthetic apparatus. Similarly, Lee and Back [91] showed that MEL interacts with ROS and RNS signaling pathways, which have been described recently as a mechanism that improves plant stress tolerance through the modulation of the activity of key antioxidant enzymes, which resulted in an alleviation in photosynthesis inhibition, and modulation of several transcription factors [91]. It has also been proven that MEL alleviates stress effects through the increase in NO levels owing to the upregulation of NR and NO synthase-related activities, also coordinating the polyamine pathway [92].

NO formation in response to abiotic stress is common in several plant species. In this sense, NO acts as a signaling molecule that regulates key responses including osmolyte accumulation, oxidative defense, photosynthesis modulation, gene expression, or PTMs of proteins [57]. NO and MEL can directly act as antioxidants by directly scavenging free radicals, alleviating oxidative damage in a receptor-independent manner [36] (Figure 2). NO is capable of balancing redox homeostasis by inducing PTMs in essential enzymes, mainly S-nitrosylation (covalent binding of NO to a cysteine thiol group) and tyrosine nitration (nitro group addition to one of the aromatic rings of tyrosine residues), as well as metal nitrosylation, which regulates its activity. These PTMs regulate the activity of the modified enzymes, especially antioxidant enzymes, but can also affect molecules such as AsA/GSH, modulating their antioxidant capacity [93]. Among the proteins regulated by NO, Ca^2+^-sensitive channels and proteins kinases are involved in the signaling cascade that leads to the adaptive response to stresses, germination, adventitious root formation, and stomatal closure [94], with the role of NO being essential for the proper functioning of these processes under stress conditions.

Recently, it has been proposed that MEL action may occur owing to a feedback mechanism modulated by H_2_O_2_ and NO, molecules which, as described previously, are essential for plant stress responses. Moreover, it is believed that interactions between NO and MEL are a necessary step for inducing the required PTMs of key stress-related proteins, which could be confirmed using proteomics analysis [37].

A plant’s exposure to temperatures above optimum leads to heat stress. Under these conditions, MEL boosts the levels of antioxidant molecules such as phenolic compounds, flavonoids (via NO-dependent pathways), and carotenoids [83,95]. In addition, in kiwifruit, Liang et al. [96] showed that exogenous MEL increased the expression of a variety of glutathione S-transferase genes, which alleviated the oxidative stress caused by high temperatures. Likewise, it has been shown that NO can also alleviate heat stress by maintaining the activity of 1,5 bisphosphate carboxylase/oxygenase (Rubisco) and enhancing photosynthetic nitrogen, and sulfur-use efficiency [97]. On the other hand, suboptimal temperatures are also harmful, and in this sense, exogenous MEL was capable of increasing the resistance against cold of plants, seeds, callus, and explants [10]. In tomatoes, MEL improved chilling tolerance by upregulating the arginine pathway, which led to higher NO levels. This helped with the maintenance of membrane integrity owing to a decrease in EL and MDA accumulation [31]. MEL treatment, in *Arabidopsis*, was capable of modulating gene expression, causing the upregulation of *CBFs, COR15a, CAMTA1*, and *ZAT10/12*, thus alleviating cold stress [98]. In tomato plants, under both heat and cold stress, MEL positively induced the activity of the arginine pathway-related enzymes, which led to higher polyamines levels and an increase in plant stress tolerance [31,92].

As is well-known, salt stress caused by excessive Na^+^ accumulation leads to osmotic stress and high ROS levels. Zhao et al. [99] showed that MEL- and NO-releasing compounds can maintain the Na^+^/K^+^ ratio during salt stress by modulating NHX1 (a sodium hydrogen exchanger) and salt overly sensitive 2 (*SOS2*) transcription levels. Thereby, NO is required for mediating MEL action in this situation [99]. The GSH/GSSG ratio is an indicator of oxidative stress, regulated by GR. During salt stress, both MEL and NO are capable of differentially regulating GR activity (thus, GSH levels), alleviating stress damage in sunflower seedlings [34,100], and revealing another point of interaction between these two molecules.

As mentioned above, under stress conditions, MEL can regulate NO levels and vice versa. Stress induced by NaCl, ZnSO_4_, and H_2_O_2_ resulted in an increase in MEL content in a time-dependent manner in barley roots [101]. In rapeseed, MEL induced NR- and NO-associated 1 (NOA1)-dependent NO formation, although it was shown that NO was not responsible for MEL synthesis and accumulation under salt stress. This condition also triggers S-nitrosylation, which was specifically induced by NO [99]. On the other hand, Arora and Bhatla [102] showed that NO was capable of reducing growth inhibition induced by salt stress in sunflower by triggering MEL accumulation, which modulated the expression of Cu/Zn SOD and MnSOD genes. Moreover, the interaction between MEL and NO also reduced the deleterious effects of salt stress by decreasing tyrosine nitration of proteins, and decreasing peroxynitrite content. As said previously, both NO and MEL can also alleviate sodic alkaline stress via the reduction of Na^+^ levels and the increase in K^+^ uptake, as well as the enhancement of antioxidant enzymes activity [82]. As water is essential for plants, the lack of it causes major damages to plants, leading to a situation known as drought stress [103]. It has been shown that exogenous MEL alleviates drought stress by increasing cell turgor, photosynthetic rate, and water-retention capacity [35]. Sharma et al. [83] demonstrated that *Carya cathayensis* plants pretreated with MEL, when subjected to drought stress, showed fewer negative effects than untreated plants. In addition, MEL upregulated primary and secondary metabolisms, such as the carotenoids pathway, under drought stress [83], and in alfalfa, MEL alleviated oxidative damage due to drought stress [35]. Likewise, MEL can also alleviate the negative effects induced by drought stress by improving plant photosynthesis. Liu et al. [104] showed that in tomato plants, exogenous MEL increased stomatal conductance, net photosynthetic rate, conductance, transpiration rate, the quantum yield of PSII, maximum quantum yield (Fv/Fm), and electron transport. Similarly, in cucumber seedlings, exogenous MEL alleviated drought effects by reducing chlorophyll degradation and increasing photosynthetic rate [105]. The rhizospheric application of MEL also improves stress tolerance. For instance, in alfalfa, Antoniou et al. [35] showed that this type of MEL application enhanced drought tolerance by regulating ROS and RNS via the modulation of SOD, GR, CAT, APX, NR, and NADH dehydrogenase activity and/or transcription. In this example, MEL application caused the downregulation of NR, decreasing NO levels, thus revealing the association of drought tolerance with reduced NO accumulation [106].

Several other studies have also shown that both NO and MEL play a role during metals’ toxicity stress response, specifically under cadmium (Cd) toxicity [107]. In *Catharanthus roseus*, MEL and sodium nitroprusside (SNP, a NO donor) improved seedling growth under Cd toxicity via the increase in the concentration of photosynthetic pigments, Cd translocation, proline concentration, and antioxidant enzymes activity (SOD, POD, APX, and CAT), leading to a decrease in lipid peroxidation and H_2_O_2_ content. Moreover, seed germination and root antioxidant response were modulated by NO, downstream of MEL [108,109,110]. Additionally, in wheat, Cd toxicity led to an increase in NO levels. Kaya et al. [111] showed that a MEL treatment was capable of enhancing Cd tolerance, with this effect reversed after cPTIO (an NO scavenger) addition, which suggested that MEL action may occur via NO increase. Conversely, under Cd toxicity, Wang et al. [112] showed that, in Chinese cabbage, NO upregulated IRT1 (a Cd-absorption-regulation-related transport gene) expression, increasing Cd absorption, which led to an intensified stress situation. However, MEL acted by inhibiting NO synthesis, thus reducing Cd levels. In terms of toxicity by other metals, as shown by Zhang et al. [113], exogenous MEL abolished NO production, alleviating aluminum-induced root growth inhibition. Meanwhile, in maize, Okant and Kaya [114] demonstrated that NO increased antioxidant enzyme activity, alleviating Pb toxicity.

Although light is essential for plants, an excessive amount of light can induce critical damage to the photosystems, resulting in what is known as high light stress. Under high light stress conditions, NO can interact with other molecules such as H_2_O_2_ (which induces stomatal closure) or inositol, mediating a situation of UV-B-initiated oxidative stress [115,116]. In maize seedlings, NO application improved high light tolerance by increasing flavonoids, anthocyanins, MDA, and UV-B-absorbing compounds’ levels, as well as increasing CAT and APX enzyme activity [117].

The climate predictions for the coming years tend to indicate adverse conditions, with devastating increases in temperature, salinity, and water scarcity. In nature, stresses do not act in an isolated manner but in combination [118], which means that plants are simultaneously subjected to two or more abiotic stresses [119]. It has been shown that the plant’s response to combined stress cannot be elucidated from the study of single stresses [120,121], and therefore, the role of NO and MEL under abiotic stress combination may be different from what has been described under single stress experiments, as described above. Recent studies by Martinez et al. [122] with tomato plants showed that, under heat and salt stress combination, MEL enhanced stress tolerance by protecting the photosynthetic apparatus and promoting ROS detoxification. Plants subjected to MEL treatment showed less lipid peroxidation and protein oxidation than untreated plants. Exogenous MEL modulated the expression of key oxidative-metabolism-related genes, such as the genes coding for APX, GR, GPX, and Ph-GPX enzymes, which led to a reduction in ROS levels [122]. MEL also modulates the concentration of osmoregulators in high-temperature stress conditions, mainly carbohydrates (such as trehalose) and amino acids (such as proline) [123]. This response was also found under stress combination (cold and drought stress) in rice plants [17]. During cold and drought stress combination, in cucumber, tomato, and tobacco, 2-hydroxymelatonin alleviated cell damage by lowering MDA production [124].

Given the few studies carried out in the field of abiotic stress combination, it is necessary to continue investigating the role of MEL and NO in the response to stress combination, and more importantly, the signaling mechanisms involved in the interaction of both molecules in light of increasing plant stress tolerance to climate change.

### 4.1. NO and MEL Interaction with Hormones during a Stress Response

Both MEL and NO interact with the main plant hormones, such as auxins (AUXs), cytokinins (CKs), ethylene, and abscisic acid (ABA), modulating the stress response. ABA is an essential phytohormone during a stress response, so its interaction with NO and MEL could provide a better understanding of the mechanisms involved in stress tolerance [125]. MEL can act via ABA signaling transduction pathways, modulating the response to salinity and drought stress, respectively [82,83]. In cucumber or apple plants, under stress situations, exogenous MEL can downregulate ABA biosynthesis and upregulate its catabolism by promoting the expression of ABA-catabolism-related genes such as *CYP707* and repressing the expression of ABA-biosynthesis-related genes such as *NCED2* or *MdNCED3* [126,127]. Li et al. [126] also proved that MEL is capable of activating ABA-mediated signaling pathways. Conversely, in water-stressed maize, exogenous MEL had no effect on ABA levels [128] and, in chilling-stressed cucumber, MEL treatment even triggered ABA production during the first 4 days [129]. As described, the effect of MEL on ABA levels is still controversial, although it is clear that MEL modulates ABA signaling transduction pathways, as MEL is capable of regulating ABA receptors, either inducing or repressing them [85,130]. ABA can also mediate NO–MEL signaling, and NO metabolism is also responsible for regulating ABA homeostasis via PTMs, which also modulates the activity of proteins from ABA-mediated signaling pathways. Moreover, NO and ABA are both capable of regulating the activity of antioxidant systems, although other molecules such as H_2_S and MEL can also be responsible for regulating these pathways [131]. Moreover, ABA can also induce PMTs during the stress response. Specifically, ABA induces S-nitrosylation of SnRK6.2/OST1 at Cys-137, inhibiting its kinase activity, which can also be induced by NO. As this kinase is part of ABA signaling pathways, these results suggest that both ABA and NO regulate ABA signaling via a negative regulatory loop [132]. Likewise, MEL, due to its auxin-like activity, is able to stimulate and modulate root generation and growth and enhance adventitious root formation, as mentioned previously [41]. Under stress conditions, the effect of MEL in stimulating plant growth is higher than under favorable conditions, as shown in salt-stressed maize or cold-stressed *Arabidopsis* plants [98,133]. There is also evidence of NO action during auxin signaling pathway activation. During Fe deficiency, in roots, Chen et al. [134] showed a correlation between auxin availability and NO levels, enhancing root ferric-chelate reductase activity, thus improving Fe uptake. Ethylene intervenes during fruit ripening, but also during the stress response. In alfalfa, MEL inhibited ethylene biosynthesis by downregulating ethylene biosynthesis-related genes. Under these conditions, MEL also promoted the accumulation of polyamines by upregulating polyamine metabolism-related enzymes, thus alleviating waterlogging stress [135]. MEL can also interact with gibberellins (GAs). Zhang et al. [127] demonstrated that cucumber seedlings treated with MEL and subjected to salt stress showed higher GA content due to the upregulation of *GA20ox* and *GA3ox*, GA biosynthesis genes, and the downregulation of ABA-biosynthesis-related genes.

During heat stress, exogenous MEL increased CK levels by upregulating *LpIPT2* and *LpOG1*, key CK-biosynthesis-related genes, while, under non-stress conditions, MEL treatment did not modify CK levels. Moreover, the CK signaling pathway was also altered by MEL via the modulation of A-ARRs and B-ARRs, transcription factors involved in CK signaling pathways [136]. NO also mediates tolerance to drought stress by modulating CK-induced photosynthetic resistance, enhancing parameters such as PSII electron donation capacity or plant photosynthetic performance index (PI). Moreover, during this response, CK increased NO synthesis via NR, which suggests that there is a crosstalk between CK and NO during the stress response [137]. To date, there have been no studies on the putative interaction of MEL and NO in the modulation or regulation of the signaling pathway of hormones. In this sense, this field of study will require special attention in the future due to, firstly, the phytohormone intrinsic characteristic of MEL and, secondly the large signaling-stress-related network governed by plant hormones. Since MEL and NO converge in many regulation points of plant hormone modulation, we speculate that it might be an interesting point of interaction between these two molecules and some key hormones.

### 4.2. Interaction of MEL or NO with H_2_S in Mediating Stress Tolerance

**Figure 3:**
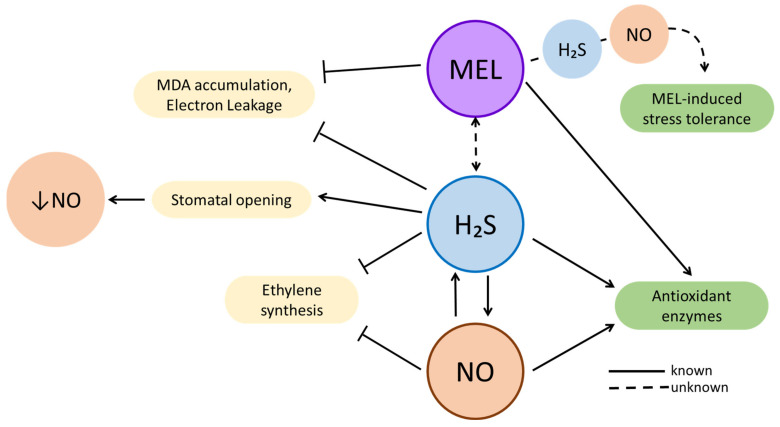
H₂S interactions with MEL or NO during abiotic stress response.

As a reactive molecule, such as ROS and NRS, hydrogen sulfide (H_2_S) is a reactive gas that requires special attention in this review, as it modulates and regulates many stress tolerance signaling pathways, fruit ripening, and cellular antioxidant enzymes’ action [138]. Therefore, given the similarity between its role and those of NO and MEL, it is interesting to mention the interactions described between H_2_S and MEL or H_2_S and NO, and between these three molecules (Figure 3).

NO and H_2_S have been reported to act both synergistically and antagonistically toward each other. H_2_S is capable of reducing NO accumulation by triggering stomatal opening [139]. However, during salt stress, H_2_S reduced oxidative damage by inducing NO production [140]. Moreover, NO was capable of alleviating hypoxia stress by triggering the activation of enzymes involved in H_2_S biosynthesis [141]. In bananas, H_2_S enhanced chilling stress tolerance by inhibiting ethylene production, a function that can also be performed by NO and MEL. It can also promote cold-stress tolerance by decreasing EL and MDA levels, as well as upregulating Ca^2+^-ATPase activity, a key secondary signaling enzyme involved in energy metabolism [142]. As shown by Li et al. [143], Cd stress induced H_2_S expression by upregulating *LCD*, *DCD*, and *DES1*. This molecule could act as a signaling molecule that modulates antioxidant enzymes, such as SOD, CAT, POD, and APX, alleviating Cd-induced oxidative stress [143]. As mentioned, both exogenous H_2_S and NO can alleviate Cd stress response. H_2_S was capable of increasing NO synthesis in alfalfa seedlings [144]. On the other hand, NO also enhanced H_2_S production in Bermuda grass and wheat [145,146]. These responses have been observed during Cd stress, which indicates that there is a NO/H_2_S crosstalk that enhances Cd stress tolerance.

Likewise, MEL can also interact with H_2_S during the abiotic stress response. In tomato cotyledons subjected to salt stress, MEL can modulate L-DES activity, thus regulating H_2_S homeostasis [147]. In addition, Siddiqui et al. [148] showed that, in tomato seedlings, MEL-mediated salt stress tolerance involves a H_2_S-dependent pathway.

Very recently, it has been suggested that stress tolerance induced by MEL might occur through a H_2_S and NO cascade. During salt stress, in pepper, both NO and H_2_S were essential for establishing MEL-induced stress tolerance. Similar results have been observed in cucumber under salt stress, pointing out that NO and H_2_S act downstream MEL during the stress response [149,150], and indicating a putative point of synergisms and interaction between these three molecules, although these results are very partial, and more investigations on their interactions and signaling mechanisms are needed.

## 5. NOmela: An Emerging Molecule with Important Stress Signaling Roles

As suggested during this review, the convergence of MEL and NO in many physiological, biochemical, and molecular events in plant cells is not fortuitous, and recently, an increased interest has been observed in a new emerging molecule with important roles in plant cells. In situations involving the presence of oxygen, at physiological pH values, MEL can be NO-nitrosated in the nitrogen atom of the indole ring, resulting in N-nitrosomelatonin (NOmela). This nitrosated form of MEL is an effective NO donor and is involved in redox signaling in plants [27,151,152,153,154,155]. The transfer of nitroso groups from N-nitrosotryptophan derivatives to MEL can also result on the formation of NOmela. This transnitrosation reaction is very strong, and it cannot be inhibited by RNS scavengers, as shown by Kirsch and De Groot [156].

**Figure 4:**
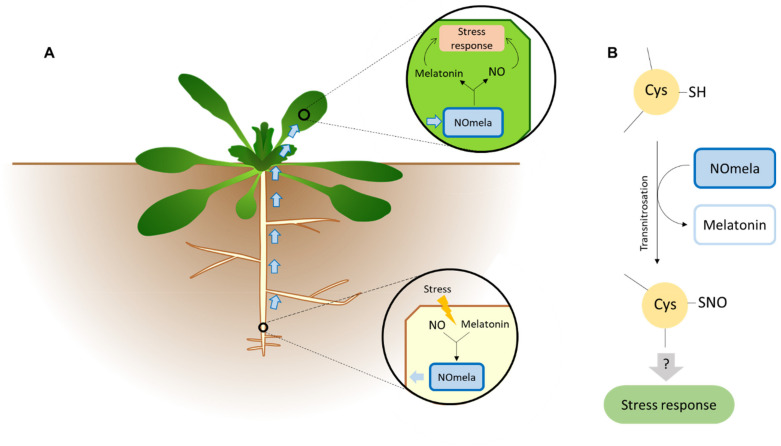
(**A**) Proposed mechanism for NO and MEL transport from roots to aerial parts via NOmela synthesis during stress conditions. Stress induces NOmela formation from NO and MEL, which can be transported to aerial parts, where it breakdowns into NO and MEL for triggering stress responses; (**B**) proposed mechanism for NOmela-mediated molecules transnitrosation mechanism under stress.

An advantage provided by NOmela is that NO release from NOmela is independent of buffer composition, but at the same time, it affects NO release from GSNO. Due to this aspect, it is believed that NOmela is a better NO precursor in cell culture. Moreover, the MEL generated by NOmela is a strong antioxidant that can protect cells in culture. This molecule is capable of releasing both NO and MEL, thus combining the beneficial proprieties of both molecules. Due to the simultaneous releasing of NO and MEL during NOmela breakdown, cells are protected from harmful effects from the RNS formed by NO autoxidation owing to MEL. In vivo, NOmela is capable of releasing NO, MEL, and SNOs without promoting the generation of hydroxyl radicals, thus avoiding their cytotoxic effects [27,154]. In mammals, NOmela has been proven to modulate circadian rhythms. The NO released from this compound was capable of enhancing its photic synchronization via increasing the immunoreactivity of key genes, as shown by Baidanoff et al. [157]. Therefore, it is possible that this compound is also capable of regulating various responses and physiological processes in other eukaryotes such as plants. In plants, NOmela is believed to exert an important role, especially under stress conditions. Roots are the first organs that are affected by soil-mediated abiotic stress. For example, sunflower seedlings showed a higher MEL concentration in roots than cotyledons after being exposed to salt stress, indicating that this organ is the first stress sensor, and therefore, MEL is rapidly synthesized. It has also been proven that abiotic stress situations can trigger long-distance signaling mediated by MEL from roots to aerial parts [158]. Likewise, due to the short half-life of NO, its transport from the roots to the aerial parts may be possible. However, this is not well-known, and further research is still needed, although it is believed that molecules such as NOmela or S-nitrosothiols such as GSNO could participate in long-distance NO transport. Moreover, in vivo studies performed by Singh et al. [159] in *Arabidopsis* seedlings showed that NOmela facilitates NO transport from roots to leaves, hence a more efficient NO donor and transporter than GSNO (Figure 4A).

As mentioned previously, NO can modify protein function or activity via PTMs; thus, we believe that NOmela can also act through this mechanism, as described in what follows. The main PTM generated by NO is a reversible redox modification called S-nitrosylation, which is characterized by the addition of a nitroso group to a thiol group present in cysteine residues (Cys) [160]. Thus, it can modulate protein stability, activity, subcellular localization, conformation, and protein–protein interactions. It is more common for S-nitrosylation to occur as a non-enzymatic process, owing to the mediation of NO, SNOs, ONNOO^−^ or higher nitrogen oxides (NO_x_), without the interplay of any co-factor or enzyme-like protein. However, transnitrosylation involves enzymes called transnitrosilases, which can transfer a nitroso group to a Cys residue [59]. This PTM is involved in important physiological processes, such as xylem vessel cell differentiation, as shown by Kawabe et al. [161]. Moreover, it can regulate plant growth and development by positively regulating auxin signaling and negatively regulating cytokinin signaling [162,163]. The plant’s response to abiotic stress is also regulated by S-nitrosylation; for instance, in response to hypoxia conditions during seed germination, GSNOR1 is subjected to S-nitrosylation, which leads to its degradation [59,164]. Tyrosine nitration is another common PTM mediated by NO, which can alter the activity of SOD, modifying ROS signaling balance [165]. During the stress response, protein arginine methylation was also modulated by NO-mediated S-nitrosylation, which indicates that cellular signaling could be regulated via interactions between different PTMs [166]. Therefore, and given the versatility of NO-inducing PTMs, NOmela may play a similar role, since NO carriers, such as LMM-SNOs, can also transnitrosate cysteine residues of proteins, altering their activity and functionality [167]. Although there are just a few studies in this field, it has been demonstrated that NOmela is capable of transnitrosating low-molecular-weight thiols, as well as vitamins and aromatic amines [156,168]. Moreover, NOmela can also react with protein thiols, which makes NOmela able to transnitrosate several proteins in their Cys residues, modulating their activity even more effectively than LMM-SNOs, as demonstrated by Kirsch and de Groot [168] (Figure 4B). Due to the importance of PTMs in regulating plant development and stress response, it is key to deepen our knowledge of NOmela’s ability to induce protein PTMs and their implications in plant physiology. From here on, we encourage the scientific community to delve into the action mechanisms of NOmela and how this attractive, novel, and very unknown molecule can act within cellular signaling mechanisms and protein activity regulation, in both animals and plants.

## 6. Conclusions and Future Perspectives

Both MEL and NO play fundamental roles in different molecular pathways present in plants. The synthesis, accumulation, transport, and action mechanisms of both molecules are fundamental—from the first stages of development to more advanced stages such as fruit ripening. In addition, they also play fundamental roles in modulating the molecular response to abiotic stresses, which requires further knowledge of the mechanisms by which this modulation occurs, to delve into the interaction of these molecules with phytohormones or with other signaling molecules such as H_2_S.

There is evidence that both molecules are capable of interacting, both at the level of biosynthesis and in more complex molecular pathways that regulate different processes, with synergistic and antagonistic interactions between them. Despite the studies presented in this review, there is still a great lack of knowledge about the exact relationship between these two molecules in numerous physiological processes, so further research is still necessary.

Given its ability to transport NO, release MEL, and induce protein PTMs, the study of NOmela promises to be a powerful source of new knowledge to better understand the molecular pathways that regulate physiological processes and stress responses in plants. In spite of this, NOmela detection tools are scarce and imprecise [154], and it is necessary to develop them correctly to properly investigate this molecule.
